# Exploring the impact of workload, organizational support, and work engagement on teachers’ psychological wellbeing: a structural equation modeling approach

**DOI:** 10.3389/fpsyg.2023.1345740

**Published:** 2024-01-19

**Authors:** Yonggang Wang

**Affiliations:** School of Public Administration, Southwestern University of Finance and Economics, Chengdu, China

**Keywords:** teacher wellbeing, workload, organizational support, work engagement, secondary school teachers, structural equation modeling, literature review

## Abstract

**Introduction:**

This study delves into the intricate relationships among workload, perceived organizational support, work engagement, and psychological wellbeing within the context of 572 secondary school teachers in China.

**Methods:**

Utilizing structural equation modeling (SEM), this research rigorously examined construct validity and the intricate interrelationships among latent variables. The data were collected and analyzed to determine the associations between workload, perceived organizational support, work engagement, and psychological wellbeing.

**Results:**

The findings unveiled compelling negative associations between workload and perceived organizational support, workload and work engagement, and workload and psychological wellbeing among the secondary school teachers. Conversely, positive correlations emerged between perceived organizational support, work engagement, and psychological wellbeing. The structural equation modeling analysis demonstrated strong fit indices, affirming robust alignment with the anticipated models.

**Discussion:**

Mediation analyses further elucidated the significance of work engagement as a mediator in the relationships between workload and psychological wellbeing, as well as between perceived organizational support and psychological wellbeing. These results underscore the pivotal role of work engagement in shaping the impact of workload and organizational support on the psychological wellbeing of secondary school teachers in Chinese educational settings.

## Introduction

In the landscape of modern education, cultivating an environment that fosters the psychological wellbeing of teachers stands as a big challenge. Teacher wellbeing encompasses a multifaceted spectrum, embracing not only emotional and professional aspects but also profoundly influencing the quality of teaching, student outcomes, and the overall educational ecosystem (Morin et al., 2017; Han et al., 2020; Zhang et al., 2023a). Within the complex set of factors shaping teacher wellbeing, three core constructs have garnered substantial attention: teacher workload, perceived organizational support, and work engagement (McInerney et al., 2015; Arslan, 2018).

Teacher workload encompasses the numerous responsibilities inherent in teaching, ranging from planning and delivering lessons to administrative duties. Research consistently highlights the burdensome nature of high workload, associating it with heightened stress levels, diminished job satisfaction, and burnout among educators (Butt and Lance, 2005; Johnson et al., 2005; Hakanen et al., 2006). Conversely, perceived organizational support signifies the degree to which teachers feel valued, appreciated, and supported by their educational institutions. Studies underline the critical role of perceived support in supporting teachers’ wellbeing by reducing burnout and enhancing job satisfaction (Eisenberger et al., 2001; Collie et al., 2015).

At the heart of this complex interplay is work engagement—an essential aspect of an educator’s professional life. Work engagement represents a state of positive fulfillment, characterized by vigor, dedication, and absorption in one’s work (Schaufeli et al., 2002). Engaged teachers exhibit resilience, passion, and a sense of purpose in their roles, ultimately shaping a favorable and productive learning environment for students. Work engagement emerges as a vital mediator within the nexus between teacher workload, organizational support, and overall wellbeing (Bakker and Demerouti, 2007; Xanthopoulou et al., 2009).

Despite the acknowledgment of these constructs, there exists a notable void in empirical investigations that concurrently explore the complex relationships between teacher workload, organizational support, work engagement, and the psychological wellbeing of teachers. This study seeks to bridge this gap by exactly examining a comprehensive model that examines the direct and mediated connections among these critical variables. By exploring these complex relationships, this research seeks to offer detailed insights into the mechanisms influencing teacher psychological wellbeing, thereby contributing to the existing body of knowledge. Additionally, the findings from this study have the potential to inform evidence-based interventions and policies aimed at fostering the psychological wellbeing and professional efficacy of educators within educational settings, ultimately enhancing the quality of education at large.

## Literature review

### Psychological wellbeing

Psychological wellbeing within the workplace is considered a pivotal determinant influencing employees’ overall satisfaction, performance, and health in their professional context (Warr, 1990; Keyes, 2002). This multidimensional construct encompasses several facets, such as feelings of competence, autonomy, positive emotions, and the absence of psychological distress (Ryff and Keyes, 1995; Deci and Ryan, 2008). Rooted in an individual’s perceptions of their work situation, it includes both hedonic (pleasure-based) and eudaimonic (meaning-based) aspects of wellbeing (Ryan and Deci, 2001; Quinn and Earnshaw, 2013).

Keyes (2002) introduced a dual-continuum model, differentiating between mental illness and mental health, highlighting that individuals can experience both mental illness (e.g., anxiety, depression) and mental health concurrently. Psychological wellbeing at work aligns closely with the mental health continuum, emphasizing positive functioning, optimal experiences, and a sense of fulfillment in the workplace (Keyes, 2002; Dagenais-Desmarais and Savoie, 2012).

Numerous studies have emphasized the significant association between psychological wellbeing at work and positive outcomes, including heightened job satisfaction, enhanced job performance, increased organizational commitment, and reduced turnover intentions (Wright and Cropanzano, 2000; Harter et al., 2002; Molero Jurado et al., 2018). Moreover, research posits a reciprocal relationship between psychological wellbeing and organizational outcomes, suggesting that improvements in wellbeing can positively impact organizational effectiveness (Judge et al., 2001). In the educational domain, particularly among teachers, psychological wellbeing holds immense importance, significantly influencing job satisfaction, motivation, and classroom effectiveness (Renshaw et al., 2015; Chitra and Karunanidhi, 2021). Given the demanding nature of the teaching profession, understanding and enhancing psychological wellbeing among educators are crucial for fostering conducive work environments and enhancing overall educational quality (Skaalvik and Skaalvik, 2017; Burić et al., 2019; Bardach et al., 2022).

Recent research has delved deeper into the complex interplay impacting mental wellness in contemporary workplaces. Fotiadis et al. (2019) explored the moderating effects of psychological self-governance, proficiency, and interconnectedness in the relationship between work-life balance and mental health. Their findings shed light on how an individual’s perception of psychological self-governance, competence, and social connections mediates the impact of work-life balance on overall mental health. Similarly, Prasad et al. (2020a) investigated remote employment during the COVID-19 pandemic in Hyderabad’s information technology sector. Their study emphasized the key role of the organizational atmosphere, opportunities, and challenges in influencing the mental wellbeing of remote workers. This investigation highlighted the significance of cultivating a supportive and adaptable organizational climate to fortify the wellbeing of remote staff.

Furthermore, Obrenovic et al. (2020) explored the complex relationship between work-family tensions, psychological safety, and mental health within job performance models. Their findings highlighted the substantial impact of work-family tensions on both psychological safety and overall mental wellness, emphasizing the imperative need to address work-family tensions to enhance employee mental wellbeing and job efficiency. Additionally, Prasad et al. (2020b) conducted an empirical analysis focusing on occupational stress, remote work, and their effects on the mental wellbeing of information technology employees. Their study revealed the detrimental impacts of occupational stress and remote employment on employees’ mental wellbeing, highlighting the urgent need for interventions to alleviate stress and support the wellbeing of remote workers.

Overall, the literature presents a comprehensive view of psychological wellbeing within the workplace, highlighting its multifaceted nature and significant impact on employee satisfaction, performance, and overall organizational effectiveness. Recent studies have further enriched our understanding, delving into various aspects of psychological wellbeing and their intricate connections within modern work environments. However, despite the extensive of research exploring psychological wellbeing in diverse contexts, a noticeable gap persists within the educational setting, particularly among Chinese teachers.

### Teacher workload

The academic exploration of teacher workload has been a main focus within the educational landscape (Smith and Bourke, 1992). This workload, including numerous instructional, administrative, and professional responsibilities (Johnson et al., 2005; Ingersoll and Strong, 2011; Chughati and Perveen, 2013), includes tasks like lesson planning, curriculum development, and classroom teaching, which have increased due to standardized testing and accountability measures (Lauermann and Karabenick, 2011; Darling-Hammond, 2017). Administrative duties, such as grading and compliance reporting, add intricacy and take over the time available for direct instructional activities (Johnson et al., 2005; Hanushek et al., 2019). Additionally, the continuous need for professional development requires teachers to stay updated with instructional advancements, further elevating their workload (Ingersoll and Strong, 2011; Opfer and Pedder, 2011). These demanding workloads have been associated with increased stress, burnout, and job dissatisfaction among teachers, potentially impacting their mental health (Maslach et al., 2001; Johnson et al., 2005; Skaalvik and Skaalvik, 2010).

In the complex link between teacher workload and psychological wellbeing, perceived organizational support (POS) stands out as a crucial factor. Teachers who perceive robust backing from their educational institutions tend to manage their workload more effectively, leading to reduced stress and burnout (Runhaar et al., 2013; Prasad et al., 2020b). Recent research has further explored the intricate relationships between teacher workload, perceived organizational support, and psychological wellbeing. Magalong and Torreon (2021) highlighted the key role of workload in shaping the holistic wellbeing of teachers across personal and professional dimensions. Pan et al. (2023) identified teacher training readiness, autonomy, and workload as key predictors of teacher wellbeing. Furthermore, Jerrim and Sims (2021) emphasized the nonlinear impact of specific teaching tasks on teacher wellbeing, emphasizing the need for a comprehensive understanding of workload components. Granziera et al. (2021) approached teacher wellbeing through the lens of the JD-R theory, providing a theoretical framework to comprehend the interplay between job demands, resources, and teacher wellbeing. Notably, Collie et al. (2015) developed the Teacher Wellbeing Scale, encompassing organizational wellbeing, and highlighted the role of perceived organizational support in shaping teacher wellbeing.

Together, these studies emphasize the multidimensional nature of factors influencing teacher wellbeing, emphasizing the complexity of teacher workload and the important role of perceived organizational support. This synthesis of findings significantly contributes to understanding the intersection between workload and organizational support, impacting the psychological wellbeing of teachers within educational contexts.

### Perceived organizational support

Perceived Organizational Support (POS) stands as an essential aspect in elucidating the complex interactions between educators and their respective educational institutions (Kurtessis et al., 2017). It encompasses employees’ perceptions of their organization valuing their contributions and caring about their wellbeing (Eisenberger et al., 1986). Within the teaching domain, POS holds significant relevance, directly influencing job satisfaction, commitment, and the overall wellbeing of teachers within their educational settings (Eisenberger et al., 2001; Rhoades and Eisenberger, 2002).

Perceived Organizational Support comprises several dimensions, including support perceived from supervisors, colleagues, and the fairness of organizational policies. Teachers feeling supported by their immediate supervisors are inclined to experience a positive work environment and heightened job satisfaction (Eisenberger et al., 1997; Rhoades and Eisenberger, 2002; Malik and Noreen, 2015). Equally crucial is the perceived support from colleagues, enhancing collaborative relationships that contribute to a positive organizational climate and influence teacher engagement and commitment (Rhoades and Eisenberger, 2002; Eisenberger et al., 2020). Furthermore, the perception of fairness in organizational policies correlates with increased commitment and job satisfaction among teachers (Rhoades and Eisenberger, 2002; Eisenberger and Stinglhamber, 2011; Chiang and Hsieh, 2012; Sudibjo and Manihuruk, 2022).

Extensive research consistently indicates a positive correlation between POS and teacher wellbeing. Teachers perceiving higher organizational support levels report lower stress, burnout, and job dissatisfaction (Eisenberger et al., 2001; Rhoades and Eisenberger, 2002). Additionally, POS acts as a protective mechanism, decreasing the negative effects of stressors, such as high workload and challenging student behaviors, on teacher psychological wellbeing (Runhaar et al., 2013; Kurtessis et al., 2017). Crucially, POS has been identified as a potential mediator in the relationship between teacher workload and wellbeing. A supportive organizational environment may decrease the negative effects of high workload on teachers, contributing to increased job satisfaction and positive work engagement (Alfes et al., 2013; Cullen et al., 2014).

An array of scholarly works has contributed to understanding the crucial role organizational support plays in shaping the psychological wellbeing of teachers. Malik and Noreen’s (2015) study investigated the complex interactions involving POS, affective wellbeing, and occupational stress, highlighting POS as a moderator in influencing the connection between affective wellbeing and occupational stress. Similarly, Journell’s (2023) doctoral dissertation scrutinized correlations among organizational support, teacher wellbeing, and resilience among secondary school educators, offering insights into the nuanced linkages between organizational support and the wellbeing and resilience of teachers.

Feni’s (2022) research focused on examining the influence of perceived organizational support and psychological capital on the psychological wellbeing of teachers. This dissertation enriched the understanding of how organizational support and psychological capital shape the psychological wellbeing of educators. Sudibjo and Manihuruk’s (2022) study explored the challenges presented by the COVID-19 pandemic, exploring how happiness at work and perceived organizational support impact teachers’ mental health through job satisfaction. This research provided valuable insights into the mediating role of job satisfaction in the relationship between happiness at work, organizational support, and mental health during tumultuous times.

Overall, these studies highlight the main influence of perceived organizational support on the psychological wellbeing of teachers. Whether moderating stress effects, contributing to resilience, or interacting with other psychological factors, organizational support emerges as a central element in nurturing the overall mental health of educators. Understanding these intricate relationships is crucial for devising interventions and policies aimed at fostering the wellbeing of teachers across diverse educational contexts.

### Work engagement

Work engagement represents a lively and positive mental state characterized by vigor, dedication, and immersion in one’s professional tasks in various domains (Schaufeli et al., 2002; Bakker et al., 2014; Pérez-Fuentes et al., 2018). In the realm of education, work engagement reflects educators’ holistic investment–physically, cognitively, and emotionally–in their professional roles (Schaufeli et al., 2006; Bakker and Demerouti, 2008; Knight et al., 2017).

The components constituting work engagement encompass vigor, dedication, and absorption. Vigor embodies high energy levels, mental resilience, and a proactive approach to work, often observed in teachers displaying enthusiasm, persistence, and zest in tackling challenges (Schaufeli et al., 2006; Li et al., 2023). Dedication involves a profound sense of significance, enthusiasm, and purpose in work, reflecting teachers’ commitment and fulfillment in contributing to students’ educational development (Schaufeli et al., 2006; Bakker and Demerouti, 2008). Absorption indicates complete immersion and focus on work, leading teachers to experience a state of flow where time becomes inconspicuous, reflecting intrinsic motivation derived from the rewards and challenges of their profession (Schaufeli et al., 2002; Bakker and Demerouti, 2008; Zhang et al., 2023b).

Extensive research consistently highlights a positive association between work engagement and teacher wellbeing (Lesener et al., 2020; Liu et al., 2023). Engaged educators are prone to experiencing heightened job satisfaction, reduced burnout, and improved psychological wellbeing (Schaufeli et al., 2006; Bakker and Demerouti, 2008; Knight et al., 2017; Fathi et al., 2023). Furthermore, the positive outcomes of work engagement extend beyond individual wellbeing, influencing the overall learning environment and ultimately impacting student outcomes (Bakker and Demerouti, 2008).

The role of POS emerges as crucial in fostering work engagement among educators. A supportive organizational environment enhances teachers’ feelings of value and engagement in their professional roles (Eisenberger et al., 2001; Bakker and Demerouti, 2008; Bakker et al., 2011; Derakhshan et al., 2023). POS serves as a catalyst, creating an atmosphere beneficial to the evolution and sustenance of work engagement among educators.

A convergence of diverse research endeavors sheds light on the intricate associations between teacher work engagement and the broader spectrum of wellbeing. For instance, Sarath and Manikandan’s (2014) exploration indicated a mutually reinforcing relationship between work engagement and the wellbeing of educators, highlighting how involvement in professional tasks intertwines intricately with teachers’ holistic wellbeing. In extending this discourse, Greenier et al. (2021) investigated the impact of emotion regulation on psychological wellbeing within the framework of work engagement among English language educators, offering insights into the emotional dimensions shaping teacher wellbeing. Zeng et al. (2019) investigated the influence of teachers’ growth mindset on work engagement within the Chinese educational context, emphasizing the role of cognitive factors in shaping teacher wellbeing. Rusu and Colomeischi (2020) explored the positivity ratio and wellbeing among teachers, highlighting the importance of positive psychological states in fostering work engagement and overall wellbeing. Han et al. (2020) scrutinized the influence of challenging job demands and resources on the wellbeing of university teachers, revealing the mediating role of teacher efficacy in the relationship between work engagement and wellbeing.

Collectively, these studies offer a comprehensive perspective on the multifaceted relationship between teacher work engagement and wellbeing. From emotional dimensions to cognitive factors, and from challenges to resources, the research presents a comprehensive view of how engagement in work contributes to the overall psychological wellbeing of educators across diverse educational settings. Understanding these intricacies is pivotal in informing interventions and strategies aimed at enhancing the wellbeing of educators.

The aim of this research is to thoroughly examine psychological wellbeing, teacher workload, perceived organizational support (POS), and work engagement in educational settings. By reviewing extensive literature in these areas, this study aims to clarify how these factors are connected and influence teachers’ psychological wellbeing. Using a structural equation modeling approach, the research seeks to understand how psychological wellbeing, workload, organizational support, and work engagement intersect and affect educators’ overall mental health and satisfaction. Table 1 summarizes the main findings related to psychological wellbeing, teacher workload, perceived organizational support, and work engagement, highlighting their connections and impact on teacher wellbeing in educational settings.

**TABLE 1:** Summary literature review.

Literature area	Key findings
Psychological wellbeing	Psychological wellbeing significantly influences workplace satisfaction, performance, and overall health (Warr, 1990; Keyes, 2002). It encompasses various aspects such as competence, autonomy, positive emotions, and absence of psychological distress (Ryff and Keyes, 1995; Deci and Ryan, 2008). This construct aligns with the mental health continuum, emphasizing positive functioning and fulfillment at work (Ryan and Deci, 2001; Quinn and Earnshaw, 2013). Linked to positive outcomes, it relates to heightened job satisfaction, enhanced performance, increased commitment, and reduced turnover intentions (Wright and Cropanzano, 2000; Harter et al., 2002; Molero Jurado et al., 2018). A reciprocal relationship exists between psychological wellbeing and organizational effectiveness (Judge et al., 2001).
Teacher workload	Teacher workload, incorporating instructional, administrative, and professional responsibilities, intensifies due to standardized testing and professional development demands (Johnson et al., 2005; Ingersoll and Strong, 2011; Chughati and Perveen, 2013). This workload associates with increased stress, burnout, and job dissatisfaction among educators, significantly impacting their mental health (Maslach et al., 2001; Johnson et al., 2005; Skaalvik and Skaalvik, 2010). Perceived organizational support (POS) plays a pivotal role in managing workload effectively, leading to stress reduction and decreased burnout (Runhaar et al., 2013; Prasad et al., 2020b).
Perceived organizational support	Perceived organizational support (POS) involves support from supervisors, colleagues, and fair organizational policies (Eisenberger et al., 1997; Rhoades and Eisenberger, 2002; Malik and Noreen, 2015). Higher POS levels correspond to lower stress, burnout, and job dissatisfaction among teachers (Eisenberger et al., 2001; Rhoades and Eisenberger, 2002). It serves as a buffer against stressors and mediates the relationship between workload and wellbeing (Alfes et al., 2013; Cullen et al., 2014).
Work engagement	Work engagement, characterized by vigor, dedication, and absorption in professional tasks, correlates with increased job satisfaction, reduced burnout, and enhanced psychological wellbeing among educators (Schaufeli et al., 2006; Bakker and Demerouti, 2008; Knight et al., 2017; Fathi et al., 2023). It positively influences the overall learning environment and student outcomes (Bakker and Demerouti, 2008). Perceived organizational support (POS) significantly contributes to fostering work engagement by creating an environment conducive to teacher involvement and fulfillment (Eisenberger et al., 2001; Bakker and Demerouti, 2008).

### The present study: hypotheses

**FIGURE 1:**
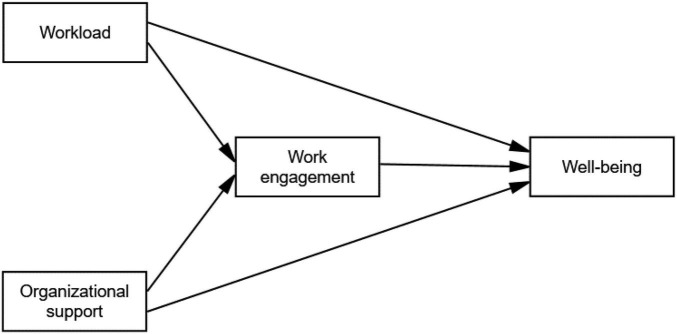
The Hypothesized Model.

In this section, we explain the hypotheses guiding our research, drawing from an extensive body of existing literature to theoretically prove and support each hypothesis in the hypothesized model (see Figure 1).

H1: *Teacher Workload is Directly Related to Teacher Wellbeing.*

The proposed hypothesis suggests a direct correlation between teacher workload and teacher wellbeing. Numerous studies have consistently highlighted the profound impact of teacher workload on their overall wellbeing (Collie et al., 2015; Granziera et al., 2021; Jerrim and Sims, 2021; Magalong and Torreon, 2021). The demands imposed on teachers, comprising both time-intensive tasks and substantial cognitive efforts, have been associated with heightened stress levels and burnout, exerting a negative influence on their wellbeing (Kyriacou, 2001; Hakanen et al., 2006; Pan et al., 2023). These findings agree with the conservation of resources theory, which posits that excessive demands, such as a high workload, may deplete an individual’s resources, consequently leading to decreased wellbeing (Hobfoll, 1989). Therefore, based on this substantial body of evidence, we hypothesize a direct and adverse relationship between teacher workload and teacher wellbeing.

H2: *Organizational Support is Directly Related to Teacher Wellbeing.*

Our second hypothesis posits a direct relationship between organizational support and teacher wellbeing. Empirical evidence consistently underscores the essential role of perceived organizational support as a determinant of teacher wellbeing (Malik and Noreen, 2015; Feni, 2022; Sudibjo and Manihuruk, 2022; Journell, 2023). When educators perceive robust support from their educational institutions, it positively influences their job satisfaction, commitment, and overall wellbeing (Eisenberger et al., 2001; Sudibjo and Manihuruk, 2022). Rooted in social exchange theory, this hypothesis suggests that employees reciprocate supportive actions with increased commitment and wellbeing (Eisenberger et al., 1986). Hence, grounded in this comprehensive empirical support, we hypothesize a direct and favorable relationship between organizational support and teacher wellbeing.

H3: *Work Engagement Mediates the Relationship Between Teacher Workload and Teacher Wellbeing.*

Drawing from the JD-R model, our third hypothesis suggests work engagement as a mediator in the relationship between teacher workload and teacher wellbeing (Bakker and Demerouti, 2007). Teacher workload, viewed as a job demand, is suggested to impact work engagement as a resource that can buffer the adverse impacts of these demands (Schaufeli and Bakker, 2004; Bakker and Demerouti, 2007). Empirical research across various occupational settings has demonstrated the mediating role of work engagement between job demands and wellbeing (Schaufeli et al., 2009; Koroglu and Ozmen, 2022). Therefore, we propose that work engagement mediates the association between teacher workload and teacher wellbeing, constituting a significant facet of this interplay.

H4: *Work Engagement Mediates the Relationship Between Organizational Support and Teacher Wellbeing.*

The fourth hypothesis posits work engagement as a mediator between organizational support and teacher wellbeing. A substantial body of literature supports the notion that organizational support enhances work engagement, thereby contributing to improved wellbeing (Schaufeli et al., 2006; Bakker and Demerouti, 2008; Sarath and Manikandan, 2014; Zeng et al., 2019; Han et al., 2020; Rusu and Colomeischi, 2020; Greenier et al., 2021). Organizational support, conceptualized as a job resource, nurtures a conducive environment that fosters work engagement (Bakker and Demerouti, 2008; Xanthopoulou et al., 2009). Guided by the JD-R model, this hypothesis proposes that organizational support significantly contributes to enhanced work engagement, subsequently influencing overall teacher wellbeing (Bakker and Demerouti, 2007). Hence, we hypothesize that work engagement mediates the link between organizational support and teacher wellbeing, including a critical mechanism within this intricate relationship.

## Materials and methods

### Participants

The investigation comprised 572 teachers employed across secondary schools in the Southwestern region of China between January 2023 and June 2023. The participants were purposefully selected during educational workshops and seminars held at various academic institutions across the region. Utilizing a purposive sampling technique, researchers invited willing participants to partake in a comprehensive questionnaire survey. Before engaging in the survey, participants provided explicit written consent. The survey instrument was administered in person by the research team.

Of the total participants, there were 138 male teachers (24.1%), 425 female teachers (74.3%), and 9 individuals for whom gender data was not available. Disciplinary distribution indicated that 236 educators specialized in language arts (41.3%), 167 in mathematics (29.2%), and 169 in various other subjects such as English, science, and music (29.5%). Additionally, 18 educators did not specify their subject. Concerning tenure, 132 educators (23.1%) reported teaching for 7 years or less, 120 (21.0%) taught between 8 and 15 years, 160 (28.0%) taught within the range of 16 to 23 years, and 160 (28.0%) had more than 24 years of teaching experience. Geographically, 132 educators (23.1%) hailed from rural school settings, while 440 (76.9%) were affiliated with urban or suburban educational institutions.

### Measures

#### Workload load scale

The study employed the Workload Scale (ECT) developed by Calderón-de et al. (2018), which delves into both quantitative and qualitative aspects of workload. This scale comprises six items distributed randomly concerning their content and is presented in an ordinal format. Respondents were required to rate these items on a five-point scale: 0 (Never), 1 (Almost never), 2 (Sometimes), 3 (Quite often), and 4 (Very often: every day). A cumulative score was calculated by summing the responses to the items. A high level of internal consistency (α = 0.82) was observed in our assessment of the ECT, signifying the coherence and reliability of the scale’s items in evaluating workload components.

#### Organizational support scale

The assessment of perceived organizational support utilized the scale developed by Ling et al. (2006). This scale employed a 6-point Likert scale ranging from 1 = “Strongly oppose” to 6 = “Strongly approve.” Initially comprising 20 items and three dimensions, four items were excluded based on the reliability and validity analyses conducted before the survey. The elimination of these items was essential to preserve the scale’s reliability and validity. With a Cronbach’s alpha of α = 0.87, this scale demonstrated a strong internal coherence, affirming its reliability in evaluating support perceptions.

#### Work engagement scale

To measure work engagement among participants, the study utilized the Utrecht Work Engagement Scale (UWES) designed by Schaufeli et al. (2002). This scale encompasses three core components: “Vigor,” “Dedication,” and “Absorption.” Comprising 17 items, respondents assessed each item on a 7-point rating scale. A sample item is “At my work, I always persevere, even when things do not go well.” UWES exhibited a commendable level of internal consistency in our study, recording a Cronbach’s alpha of α = 0.89.

#### Psychological well-being scale

The assessment of psychological wellbeing at work utilized the questionnaire developed by Dagenais-Desmarais and Savoie (2012), known as the Psychological Wellbeing at Work (PWBW) inventory. This instrument measures five main dimensions: “Interpersonal Fit at Work,” “Thriving at Work,” “Feeling of Competency at Work,” “Perceived Recognition at Work,” and “Desire for Involvement at Work.” Comprising 25 items, respondents rated each item on a 6-point scale, ranging from 0 = Disagree to 5 = Completely Agree. A sample item includes “I feel that my work efforts are appreciated.” The PWBW inventory showed high internal reliability (α = 0.88) in our analysis, ensuring consistent measurement of wellbeing facets among respondents.

### Data analysis

The data analysis process encompassed SPSS version 28.0 and AMOS version 26.0 for comprehensive examination. Initially, confirmatory factor analysis (CFA) was employed to assess the construct validity of each scale utilized in the study (Brown, 2006). Subsequently, descriptive statistics were computed, including mean (M) and standard deviation (SD), while correlations among variables were determined using SPSS.

To test the formulated hypotheses, the structural equation modeling (SEM) approach was utilized, along with mediation analysis. The assessment of model fit employed several indices, namely the chi-square statistic (χ2), root mean square error of approximation (RMSEA), Tucker–Lewis index (TLI), and comparative fit index (CFI). For determining acceptable data fit, the study employed the criteria suggested by Hu and Bentler (1999), which considered a combination of CFI > 0.90, TLI > 0.90, and RMSEA < 0.1 as the cutoff thresholds.

Moreover, to ascertain mediation effects, a bootstrapping method following Hayes (2009) was employed. This technique was instrumental in detecting and estimating the significance of mediation effects within the structural model.

## Results

Descriptive statistics for the study variables, as well as their correlations, are presented in Table 2. The mean and standard deviation (SD) for workload, organizational support, work engagement, and wellbeing were 4.02 (0.58), 3.10 (0.89), 3.78 (0.70), and 4.20 (0.65), respectively.

**TABLE 2:** Descriptive statistics and correlations.

Variables	Mean (SD)	1	2	3	4
1. Workload	4.02 (0.58)	1.00			
2. Organizational support	3.10 (0.89)	-0.24*	1.00		
3. Work engagement	3.78 (0.70)	-0.18*	0.42**	1.00	
4. Wellbeing	4.20 (0.65)	-0.35**	0.54**	0.48**	1.00

*p < 0.05. **p < 0.01.

Regarding the correlations, workload demonstrated a significant negative association with organizational support (*r* = −0.24, *p* < 0.05), as did workload with work engagement (*r* = −0.18, *p* < 0.05) and workload with wellbeing (*r* = −0.35, *p* < 0.01). Notably, organizational support exhibited a positive and significant correlation with work engagement (*r* = 0.42, *p* < 0.01) and wellbeing (*r* = 0.54, *p* < 0.01). Additionally, a positive correlation was observed between work engagement and wellbeing (*r* = 0.48, *p* < 0.01). These findings suggest significant associations among the variables, highlighting the interconnected nature of workload, organizational support, work engagement, and psychological wellbeing among Chinese teachers.

Following the preliminary data screening, Confirmatory Factor Analysis (CFA) was deployed to appraise the construct validity inherent in the measurement models. In assessing the adequacy of these models, various indices indicative of goodness of fit were employed. These measurement models encompassed latent constructs such as workload, organizational support, work engagement, and psychological wellbeing.

Initially, upon analysis, certain measurement models displayed inadequate fit to the collected data. Consequently, adjustments were undertaken to enhance their congruence with the empirical data. To achieve this, a strategic alteration approach was adopted. Specifically, three items from the wellbeing scale and two items from the work engagement scale were removed owing to their lower factor loadings, falling below the threshold of 0.40. Furthermore, two correlational pathways were introduced between error terms associated with two constructs, namely workload and organizational support. Following these adjustments, the refined and modified measurement models demonstrated a notable improvement, exhibiting satisfactory alignment with the collected dataset. Detailed statistical summaries and model fit indices are provided in Table 3 for comprehensive review and assessment of the refined models.

**TABLE 3:** Measurement model of the latent variables.

Latent variables	*χ^2^*	df	χ^2^/df	CFI	TLI	RMSEA
Workload	315.80	147	2.15	0.94	0.94	0.05
Organizational Support	152.25	72	2.11	0.93	0.94	0.05
Work Engagement	271.50	130	2.09	0.94	0.93	0.04
Wellbeing	395.60	200	1.98	0.96	0.95	0.03

In order to establish convergent validity, the Average Variance Extracted (AVE) was employed, aligning with the methodology outlined by Fornell and Larcker (1981). The evaluation, detailed in Table 4, demonstrates that both the AVE and Construct Reliability (CR) for the constructs surpassed the recommended threshold values of 0.50 and 0.60, respectively. This outcome suggests robust convergent validity. Notably, all indicators within the foundational measurement model exhibited loadings greater than 0.5, serving as compelling evidence affirming the convergent validity of the constructs.

**TABLE 4:** Convergent and discriminant validity.

	AVE	CR	1	2	3	4
Self-efficacy	0.62	0.89	0.79			
Emotion regulation	0.58	0.91	-0.24*	0.76		
Resilience	0.53	0.83	-0.18*	0.42**	0.73	
Burnout	0.72	0.86	-0.35**	0.54**	0.48**	0.85

AVE, average variance extracted; CR, composite reliability. The diagonal line values are the square root of AVE. The off-diagonal line values are the correlation coefficients of one factor with another factor. *p < 0.05, **p < 0.01.

Furthermore, discriminant validity was rigorously evaluated employing Straub et al.’s (2004) recommended criterion. This involved an examination comparing the square root of AVE with the correlation coefficient between related constructs. The findings, as illustrated in Table 4, unveiled that the interrelationships among all factors remained notably lower than the square root of the respective AVE values. This outcome validates the discriminant validity, affirming that the constructs are distinct from each other and can be differentiated effectively within the measurement model.

Following the hypothesized relationships among the latent variables, SEM was employed to investigate these associations. The findings unveiled a robust alignment between the anticipated model and the actual dataset, showcasing noteworthy fit indices: *χ^2^* = 660.120, df = 450, *p* < 0.001, CFI = 0.981, TLI = 0.972, RMSEA = 0.025 (95% CI [0.020, 0.030]), and SRMR = 0.042.

**FIGURE 2:**
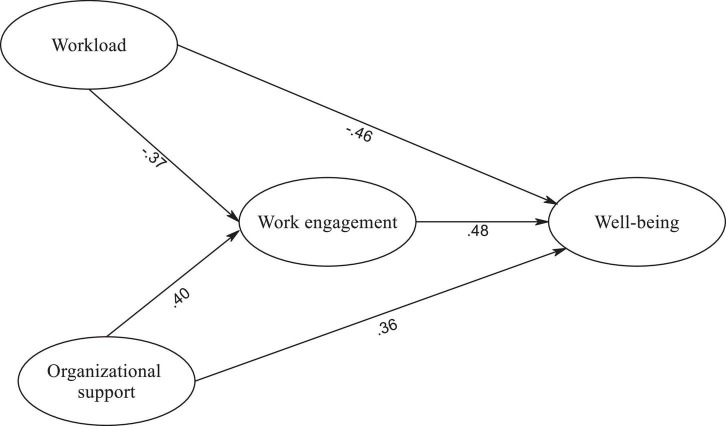
The Mediation Model.

Illustrating the envisioned connections between the latent constructs, Figure 2 exhibits the path diagram representing these relationships. Notably, all path coefficients emerged as statistically significant, affirming and providing substantial support for the anticipated associations between the variables.

Finally, to ascertain the significance of indirect effects, 5000 resamples bootstrapping analyses were conducted following Hayes’s (2009) method. Table 5 presents a comprehensive overview of the direct, indirect, and total effects observed in the mediation analysis.

**TABLE 5:** The results of mediation analysis.

Path	β	95% CI	T statistics	*p*
Direct effects				
Workload → wellbeing	0.46	[0.41, 0.52]	12.60	<0.001
Organizational support → wellbeing	0.36	[0.31, 0.42]	9.80	<0.001
Work engagement → wellbeing	0.48	[0.43, 0.54]	13.40	<0.001
Indirect effects				
Workload → WE → wellbeing	0.17	[0.13, 0.22]	6.20	<0.001
Organizational support → WE → wellbeing	0.19	[0.15, 0.24]	7.00	<0.001
Total effects				
Workload → wellbeing (Total)	0.63	[0.58, 0.68]	18.50	<0.001
Organizational support → wellbeing (Total)	0.55	[0.50, 0.60]	16.20	<0.001

WE, work engagement. Bootstrap is based on 5000 resamples (Hayes, 2009).

Notably, direct effects of workload, organizational support, and work engagement on wellbeing were statistically significant (workload → wellbeing: β = 0.46, 95% CI [0.41, 0.52], *T* = 12.60, *p* < 0.001; organizational support → wellbeing: β = 0.36, 95% CI [0.31, 0.42], *T* = 9.80, *p* < 0.001; work engagement → wellbeing: β = 0.48, 95% CI [0.43, 0.54], *T* = 13.40, *p* < 0.001).

Additionally, indirect effects were observed, highlighting the mediating role of work engagement in the relationships between workload and wellbeing (workload → work engagement → wellbeing: β = 0.17, 95% CI [0.13, 0.22], *T* = 6.20, *p* < 0.001) and between organizational support and wellbeing (organizational support → work engagement → wellbeing: β = 0.19, 95% CI [0.15, 0.24], *T* = 7.00, *p* < 0.001).

Furthermore, the combined direct and indirect pathways, termed as total effects, exhibited substantial influence (workload → wellbeing (Total): β = 0.63, 95% CI [0.58, 0.68], *T* = 18.50, *p* < 0.001; organizational support → wellbeing (Total): β = 0.55, 95% CI [0.50, 0.60], *T* = 16.20, *p* < 0.001).

Overall, the mediation analysis revealed indirect effects, highlighting the mediating role of work engagement in the relationships between workload and psychological wellbeing, as well as between organizational support and psychological wellbeing. This suggests that work engagement plays a crucial intermediary role in influencing the impact of workload and organizational support on teachers’ psychological wellbeing.

## Discussion

This study thoroughly studied the complex interrelationships among teacher workload, organizational support, work engagement, and psychological wellbeing within the setting of Chinese secondary schools. The findings discovered important information on the complex relationships that influence educators’ psychological health, shedding profound light on how work demands and support systems intertwine to impact teachers’ overall wellbeing.

The observed direct correlation between teacher workload and wellbeing resonates deeply with an extensive body of literature underscoring the harmful impacts of heightened work demands on teachers’ mental health (Hakanen et al., 2006; Skaalvik and Skaalvik, 2010; Collie et al., 2015; Granziera et al., 2021; Jerrim and Sims, 2021; Magalong and Torreon, 2021; Pan et al., 2023). The inherent responsibilities entailed in teaching, including multifaceted tasks such as lesson planning, grading, and administrative duties, notably contribute to escalated stress and burnout among educators (Kyriacou, 2001; Granziera et al., 2021). These findings align harmoniously with the conservation of resources theory, postulating that excessive demands can deplete an individual’s resources, potentially jeopardizing their overall wellbeing (Hobfoll, 1989). As educators navigate the complexities of their profession, the pressure caused by high workload emerges as a pivotal and influential factor that significantly impacts their psychological health and overall wellbeing (Jerrim and Sims, 2021).

Implementing targeted interventions to address workload concerns, such as optimizing administrative processes or ensuring adequate resource provisions, not only holds the potential to enhance wellbeing but also emerges as a facilitator for boosting job satisfaction and increasing teacher retention rates (Ingersoll and Strong, 2011; Schleicher, 2018). This study underscores the high importance of addressing workload as a modifiable factor to enhance teacher wellbeing and consequently, elevate the quality of education (Skaalvik and Skaalvik, 2010; Collie et al., 2015; Magalong and Torreon, 2021).

Also, the direct correlation between organizational support and teacher wellbeing harmonizes with a rich body of empirical research emphasizing the profound impact of supportive work environments on teachers’ mental health (Malik and Noreen, 2015; Feni, 2022; Sudibjo and Manihuruk, 2022; Journell, 2023). A conducive work atmosphere, where educators perceive organizational support and recognition for their wellbeing, cultivates a positive environment, leading to increased job satisfaction and reduced burnout (Eisenberger et al., 2001; Malik and Noreen, 2015). This congruence aligns inherently with social exchange theory, postulating that perceived organizational support generates increased commitment and wellbeing among employees (Eisenberger et al., 1986). The current study contributes empirical evidence, further bolstering the direct link between organizational support and teacher wellbeing.

Additionally, the identified mediating function of work engagement in the interplay between teacher workload and wellbeing substantiates the JD-R model, emphasizing work engagement as a critical mediator between job demands and wellbeing (Bakker and Demerouti, 2017). High levels of work engagement are posited as invaluable resources that lessen the detrimental effects of workload demands (Bakker and Demerouti, 2017; Montani et al., 2020). This finding resonates with existing research in diverse occupational contexts, signifying that nurturing work engagement is pivotal not only for individual wellbeing but also for fostering a thriving and effective workforce (Schaufeli et al., 2009; Sarath and Manikandan, 2014; Han et al., 2020). In the realm of teaching, characterized by intrinsic workload demands, cultivating work engagement surfaces as a crucial element, indispensable for not only individual teacher wellbeing but also for nurturing a positive and effective teaching workforce (Schaufeli et al., 2006; Bakker and Demerouti, 2008; Rusu and Colomeischi, 2020).

Moreover, the study highlights the mediating role of work engagement between organizational support and wellbeing, aligning consistently with the JD-R model. Perceived organizational support profoundly influences educators’ work engagement, fostering a constructive work-related state (Eisenberger et al., 2001; Xanthopoulou et al., 2009; Sudibjo and Manihuruk, 2022; Journell, 2023). Particularly in the context of teachers, organizational support emerges as a pivotal driver in promoting work engagement, serving as a protective mechanism against stress and workload (Sudibjo and Manihuruk, 2022). Educators perceiving heightened levels of organizational support tend to exhibit increased dedication, vigor, and absorption in their professional roles, resulting in heightened engagement (Schaufeli et al., 2002; Malik and Noreen, 2015).

The ramifications of these findings hold considerable weight for educational policymakers and administrators striving to elevate teacher wellbeing and consequently, the quality of education. Initiatives aimed at fostering a supportive organizational climate, including the implementation of mentorship programs, offering professional development opportunities, and establishing transparent communication channels, emerge as pivotal strategies in nurturing work engagement among teachers (Eisenberger et al., 2001; Collie et al., 2015). These interventions, besides contributing to immediate wellbeing, wield the potential to significantly enhance instructional quality and yield positive outcomes for student education (Schaufeli et al., 2009; Collie et al., 2015). Furthermore, recognizing the intricate interdependence between organizational support and work engagement underscores the amplified positive effects of organizational support on teacher wellbeing, creating a reinforcing cycle that mutually benefits educators and educational institutions (Bakker and Demerouti, 2008).

## Conclusion

This study’s exploration into the multifaceted factors shaping teacher wellbeing within educational settings has unraveled critical insights into the complex interconnections among teacher workload, organizational support, work engagement, and overall teacher wellbeing. The findings underscore robust direct relationships among these elements, offering insights into how workload, organizational support, and work engagement intricately influence and shape teacher wellbeing. The identified direct impact of teacher workload emphasizes the pressing necessity for targeted interventions aimed at reducing the burdens on educators. Policymakers and school administrators can implement focused initiatives, such as streamlining administrative processes and ensuring sufficient resources, which stand as potential contributors to sustaining the wellbeing of the teaching workforce.

Moreover, perceived organizational support serves as a fundamental catalyst in enhancing the wellbeing of secondary school teachers in China. By “catalyst”, we mean that POS acts as a crucial agent that speeds up and strengthens the positive effects of supportive environments on teachers’ wellbeing. It plays a pivotal role in mediating the impact of workload and making the positive effects of supportive environments stronger for teacher wellbeing. In other words, POS plays a pivotal role in mediating the impact of workload and amplifying the positive effects of supportive environments on teacher wellbeing. This study’s identification of POS as a catalyst contributes significantly to understanding how organizational support directly influences teachers’ work engagement and psychological wellbeing. This insight highlights the importance of supportive cultures within educational institutions and provides a framework for future interventions and policies aimed at enhancing teacher wellbeing and, subsequently, educational effectiveness. This identified catalyst role of POS also holds substantial significance for future researchers. Understanding POS as a catalyst offers a framework to explore and design interventions that specifically target organizational support mechanisms within educational settings. Future researchers can utilize this understanding to develop nuanced strategies, interventions, and policies aimed at enhancing teacher wellbeing. By focusing on fostering supportive cultures and improving organizational support, researchers can contribute to refining the educational environment, ultimately benefiting both educators and students. Furthermore, acknowledging POS as a catalyst opens avenues for exploring and evaluating the effectiveness of various interventions, enabling a more tailored approach to improving teacher wellbeing and, consequently, the quality of education.

Furthermore, the highlighted mediating role of work engagement emphasizes the potential for interventions that elevate teacher engagement to act as both a shield against the adverse effects of high workload and an amplifier of the positive impacts of organizational support. Implementation of strategies like professional development programs, mentorship initiatives, and recognition for exemplary work can serve as pivotal mechanisms in enhancing engagement among teachers.

The implications of this study resonate profoundly with educational policymakers, school administrators, and practitioners involved in teacher development. Recognizing the direct impact of teacher workload underscores the urgency for systemic changes aimed at reducing undue burdens on educators. Concurrently, interventions aimed at reinforcing organizational support can significantly mold supportive work environments conducive to teacher wellbeing.

Moreover, these findings go beyond the realm of teacher welfare, extending to broader implications for the quality of education. An engaged teaching workforce is likely to create positive learning environments, ultimately influencing student outcomes and enhancing educational effectiveness. Consequently, investing in teacher wellbeing emerges as an integral component of broader educational enhancement initiatives.

However, acknowledging the study’s contributions, several limitations warrant consideration. The cross-sectional nature of the data hinders definitive establishment of causality. Longitudinal studies could provide a more intricate understanding of the evolving relationships among teacher workload, organizational support, work engagement, and wellbeing over time. Additionally, reliance on self-report measures introduces the potential for common method bias. Future research incorporating objective measures and diverse data sources could fortify the robustness of the findings. Moreover, the study’s generalizability may be influenced by contextual and cultural factors within the educational system, necessitating replication across diverse settings to enhance external validity.

Taken together, while this study lays a solid groundwork for comprehending the intricate dynamics influencing teacher wellbeing, it serves as a catalyst for future research endeavors. By informing and inspiring practical interventions aimed at enhancing teacher wellbeing, this study contributes profoundly to the holistic enhancement of the educational ecosystem.

## Data availability statement

The raw data supporting the conclusions of this article will be made available by the authors, without undue reservation. Requests to access these datasets should be directed to YW, wangyonggang1920@sina.com.

## Ethics statement

The studies involving humans were approved by the School of Public Administration, Southwestern University of Finance and Economics, Chengdu, China. The studies were conducted in accordance with the local legislation and institutional requirements. The participants provided their written informed consent to participate in this study.

## Author contributions

YW: Conceptualization, Data curation, Formal analysis, Investigation, Methodology, Project administration, Resources, Software, Supervision, Validation, Visualization, Writing—original draft, Writing—review and editing.
